# Cellular signatures in human blood track bone mineral density in postmenopausal women

**DOI:** 10.1172/jci.insight.178977

**Published:** 2024-11-22

**Authors:** Kaichi Kaneko, Jefferson Tsai, Deniece Meñez, Brian Oh, Andrew Junwoo Suh, Seyeon Bae, Masataka Mizuno, Akio Umemoto, Eugenia Giannopoulou, Takayuki Fujii, Yaxia Zhang, Emily M. Stein, Richard S. Bockman, Kyung-Hyun Park-Min

**Affiliations:** 1 Arthritis and Tissue Degeneration Program, David Z. Rosensweig Genomics Research Center, Hospital for Special Surgery, New York, New York, USA.; 2 Division of Rheumatology, Department of Internal Medicine, Toho University Sakura Medical Center, Sakura, Chiba, Japan.; 3 SUNY Downstate Health Sciences University, Brooklyn, New York, USA.; 4 Biological Sciences Department, New York City College of Technology, City University of New York, Brooklyn, New York, USA.; 5 Pathology and Laboratory Medicine, Hospital for Special Surgery, New York, New York, USA.; 6 Pathology and Clinical Laboratory Medicine, Weill Cornell Medical College, New York, New York, USA.; 7 Endocrine Service, Hospital for Special Surgery, New York, New York, USA.; 8 Department of Medicine, Weill Cornell Medical College, New York, New York, USA.; 9 BCMB Allied Program, Weill Cornell Graduate School of Medical Sciences, New York, New York, USA.

**Keywords:** Bone biology, Osteoclast/osteoblast biology, Osteoporosis

## Abstract

Osteoclasts are the sole bone-resorbing cells and are formed by the fusion of osteoclast precursor cells (OCPs) derived from myeloid lineage cells. Animal studies reveal that circulating OCPs (cOCPs) in blood travel to bone and fuse with bone-resident osteoclasts. However, the characteristics of human cOCPs and their association with bone diseases remain elusive. We have identified and characterized human cOCPs and found a positive association between cOCPs and osteoclast activity. Sorted cOCPs have a higher osteoclastogenic potential than other myeloid cells and effectively differentiate into osteoclasts. cOCPs exhibit distinct morphology and transcriptomic signatures. The frequency of cOCPs in the blood varies among treatment-naive postmenopausal women and has an inverse correlation with lumbar spine bone density and a positive correlation with serum CTX, a bone resorption marker. The increased cOCPs in treatment-naive patients with osteoporosis were significantly diminished by denosumab, a widely used antiresorptive therapy. Our study reveals the distinctive identity of human cOCPs and the potential link between the dynamic regulation of cOCPs and osteoporosis and its treatment. Taken together, our study enhances our understanding of human cOCPs and highlights a potential opportunity to measure cOCPs through a simple blood test, which could potentially identify high-risk individuals.

## Abstract 2

A specific subset of blood cells serves as a precursor to osteoclasts, bone resorbing cells, and is associated with changes in bone density in postmenopausal women.

## Graphical abstract

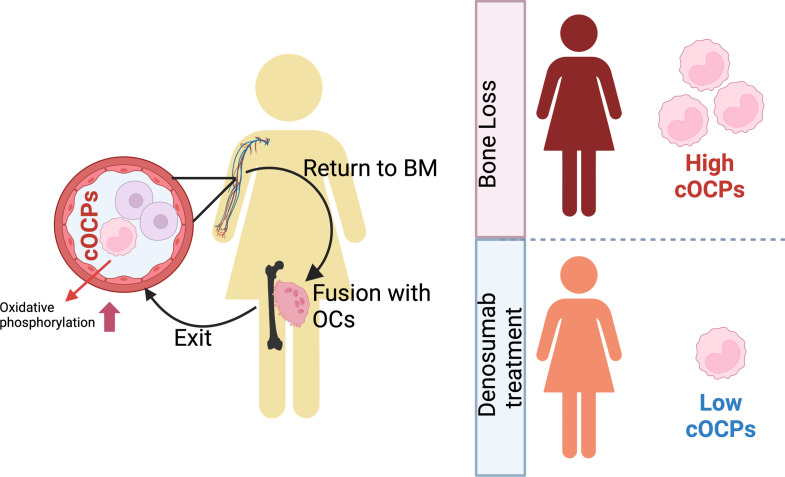

## Introduction

Osteoporosis is a systemic bone disorder causing low bone mass and compromised bone strength, leading to an increased risk of fracture (1–3). Osteoporosis is a silent disease that often remains undetected until a fracture occurs owing to the absence of noticeable symptoms (4). Since effective osteoporosis treatment options are available, osteoporosis can be managed. However, many high-risk individuals have never been screened, and the treatment of osteoporosis is delayed until the proper diagnosis. The current diagnosis of osteoporosis and assessment of fracture risk are based on the quantitative analysis of bone mineral density (BMD) by dual-energy x-ray absorptiometry (DXA), which is underutilized (5). Thus, the efficient identification of high-risk individuals is essential and necessary.

Osteoclasts are the sole bone-resorbing cells (6–10) and are derived from myeloid lineage cells, termed osteoclast precursor cells (OCPs) (11). The RANKL and macrophage colony-stimulating factor (M-CSF) are key factors for osteoclastogenesis. It has been shown that hematopoietic stem cell transplantation or infusion of circulating cells via parabiosis can improve bone phenotype in humans with osteopetrosis and osteopetrotic mice (11, 12). Another study demonstrated that circulating OCPs (cOCPs) travel to bone and fuse with bone-resident osteoclasts (11), suggesting that circulating cells contain OCPs and contribute to the maintenance of osteoclasts. Different subsets of OCPs have been identified in bone marrow, blood, or metastatic bone lesions by the expression of different cell surface markers, which are commonly used for defining myeloid cells. However, distinct OCP-specific markers remain inconclusive, and their characteristics have not been fully determined.

In this study, we have identified a subset of CD14^+^ cells with a high potential to differentiate into osteoclasts (referred to as cOCPs). cOCPs are distinct from other CD14^+^ monocytes in terms of their cellular morphology and transcriptomic profile. cOCPs express genes related to MYC targets and metabolic pathways. Notably, the frequency of cOCPs in blood is inversely correlated with bone density in postmenopausal women. Blocking RANKL by denosumab, an FDA-approved anti-RANKL antibody, has effectively improved bone density and fracture risk in patients with osteoporosis (13, 14). We found that increased cOCPs in postmenopausal women with osteoporosis were significantly diminished by denosumab treatment. Our study suggests that cOCPs could serve as a valuable tool for identifying individuals at high risk of pathological bone loss and as a potential therapeutic target for bone diseases.

## Results

### A RANKL-responsive subset of CD14^+^ monocytes.

**Figure 1:**
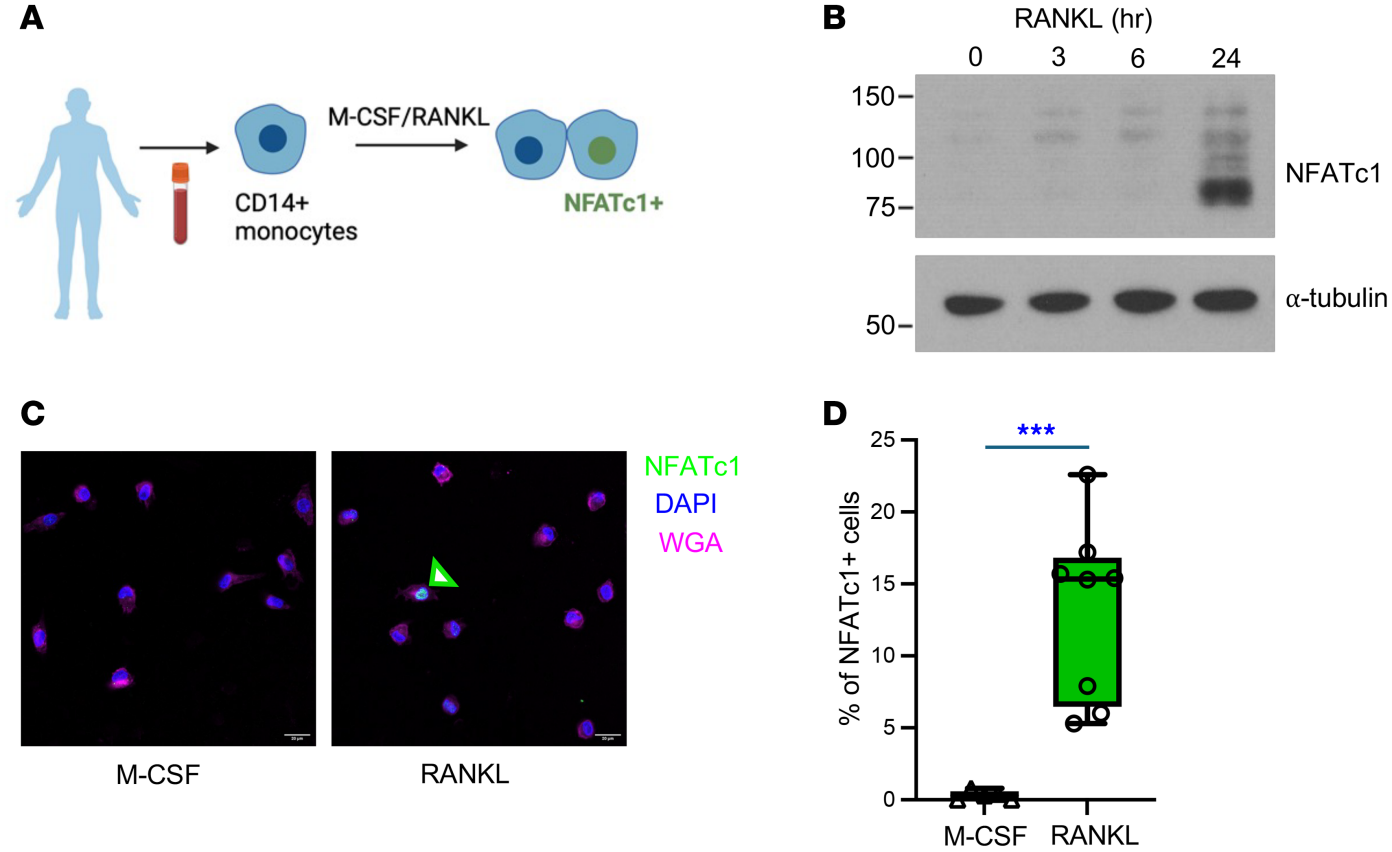
A subset of CD14^+^ cells shows a distinct response to RANKL. (**A**) Schematic for RANKL-induced NFATc1 activation. (**B**–**D**) CD14^+^ cells were treated with M-CSF or M-CSF and RANKL for the indicated times. (**B**) Total lysates were analyzed by immunoblot using anti-NFATc1 antibodies and α-tubulin antibodies as a loading control (*n* = 3). Values are shown in kDa. (**C** and **D**) Cells were treated with RANKL for 24 hours and were stained with anti-NFATc1 antibodies, DAPI (blue) to visualize the nucleus, and wheat germ agglutinin (WGA, pink) to visualize intracellular vesicles and membranes. (**C**) Representative images of NFATc1-stained cells (*n* ≥ 5). Scale bar: 20 μm. (**D**) Quantification of NFATc1^+^ cells. All data are shown as median and interquartile range. ***P < 0.005 by 2-tailed Student’s *t* test.

Human circulating CD14^+^ cells, known as monocytes, can differentiate into osteoclasts in vitro upon treatment with RANKL, a key factor for osteoclastogenesis (15–17). CD14^+^ cells are an essential subtype of the innate immune system (18). However, CD14^+^ cells are now considered heterogeneous populations based on their transcriptomic profiles (19). We and others showed that RANKL stimulation activates NFATc1, a master transcription factor of osteoclastogenesis, initiating the osteoclastogenic program (20). The expression of NFATc1 protein was measured in CD14^+^ cells after RANKL stimulation using immunoblot and immunofluorescence analysis (Figure 1A). As expected, NFATc1 protein in total lysates was induced by RANKL stimulation (Figure 1B). However, immunofluorescence analysis showed that the percentage of NFATc1^+^ cells significantly increased in RANKL-treated CD14^+^ cells compared with the control and was detected in only a subset of CD14^+^ cells (Figure 1, C and D). To corroborate our findings, we analyzed NFATc1 mRNA expression in CD14^+^ cells using single-cell RNA-Seq. Accordingly, RANKL stimulation increased the expression of NFATc1 mRNA in a subset of CD14^+^ cells (Supplemental Figure 1; supplemental material available online with this article; https://doi.org/10.1172/jci.insight.178977DS1), supporting that a subset of CD14^+^ cells preferentially respond to RANKL at a higher rate than other subsets.

### cOCPs have a high osteoclastogenic potential.

**Figure 2:**
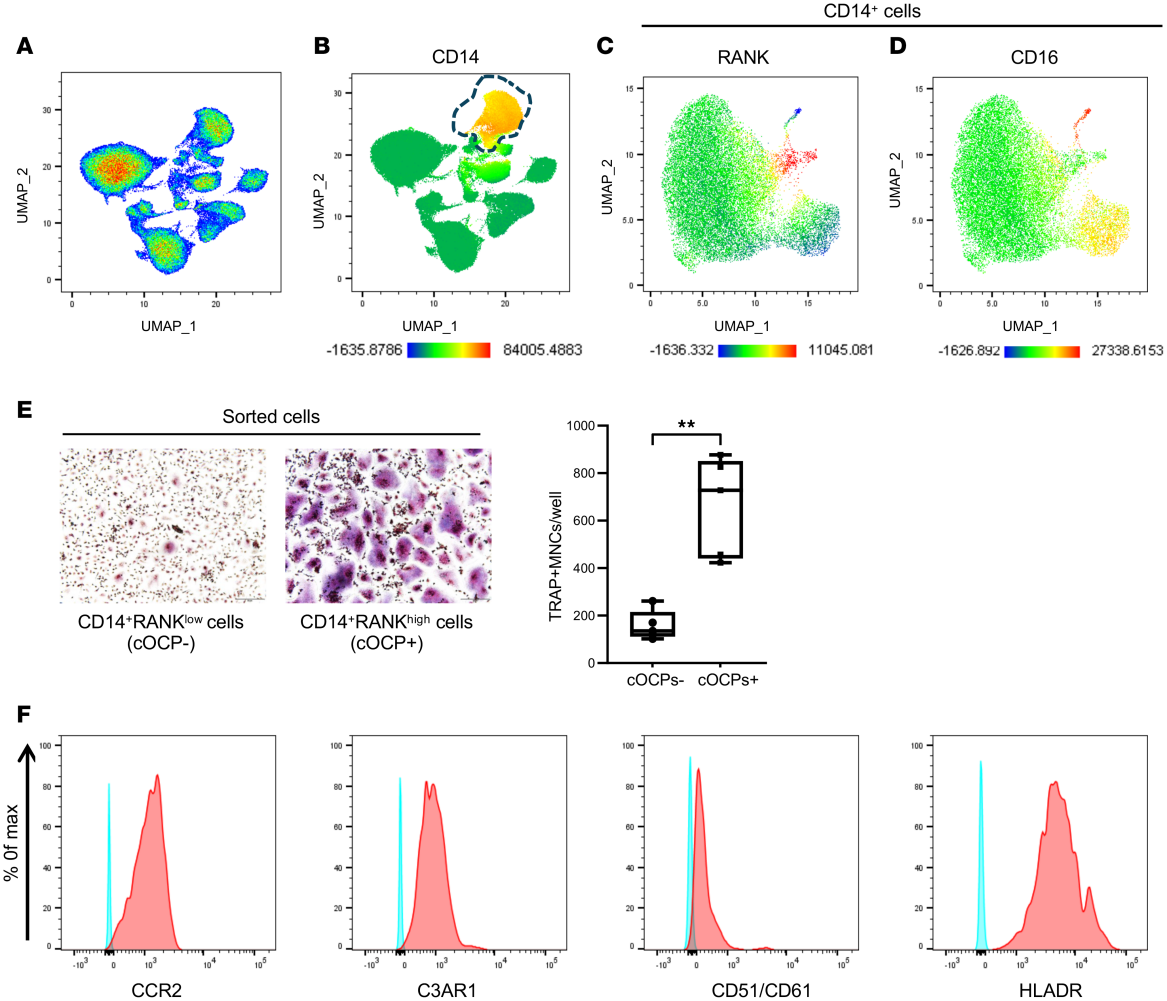
Analysis of circulating osteoclast precursor cells. (**A**–**D**) UMAP analysis of human PBMCs. Human PBMCs from healthy donors were stained and analyzed with flow cytometry (*n* = 9). (**A**) UMAP plot showing the different cell populations of PBMCs. (**B**) UMAP plot, with color coding (red to blue) for the expression of CD14, a marker gene of human monocytes. (**C** and **D**) UMAP plot of CD14^+^ cells for the expression of RANK (**C**) and CD16 (**D**). (**E**) Osteoclastogenesis assay (*n* = 5). Osteoclast differentiation in CD14^+^ cells with or without OCPs. TRAP^+^ multinucleated cells (MNCs, ≥ 3 nuclei) were counted as osteoclasts in triplicate. The left panel shows representative images. The right panel shows the number of TRAP^+^ MNCs. All data are shown as median and interquartile range. **P < 0.01 by 2-tailed, unpaired *t* test. (**F**) Representative histograms of CCR2, C3AR1, CD51/CD61, and HLA-DR expression in cOCPs using cumulative data pooled from 3 independent donors.

RANK, a receptor for RANKL, is required for the response to RANKL (20, 21). We examined the RANK expression of CD14^+^ cells. We applied multiparameter flow cytometry analysis with a 16-antibody flow panel and visualized different subsets of PBMC by a computational approach (22) and uniform manifold approximation and projection (UMAP) for dimensionality reduction (23) (Figure 2A and Supplemental Figure 2A). The CD14^+^ population was clearly separated from other cells in PBMCs and was defined as CD45^+^Lin^–^ (negative for T cells, B cells, NK cells, and red blood cells) (Figure 2B and Supplemental Figure 2B). CD16 has been used to classify a disease-associated subset of monocytes. Using antibodies against RANK and CD16, we found a subset of CD14^+^ cells expressing RANK or CD16 (Figure 2, C and D). However, these two subsets did not overlap (Figure 2, C and D). Consistently, RANK was also expressed on a subset of CD14^+^ cells after M-CSF treatment (Supplemental Figure 3).

To further test the osteoclastogenic potential of CD14^+^ subpopulations, both sorted CD14^+^CD16^+^ cells and CD14^+^RANK^hi^ cells were subjected to osteoclastogenesis assay. Sorted CD14^+^CD16^+^ cells or CD14^+^ RANK^hi^ cells were cultured with M-CSF and RANKL to form TRAP^+^ multinuclear osteoclasts. CD14^+^RANK^hi^ cells or CD14^+^CD16^–^ cells formed osteoclasts, while CD14^+^RANK^lo^ cells or CD14^+^CD16^+^ cells showed very low osteoclastogenic potential (Figure 2E and Supplemental Figure 4, A and B), supporting that CD14^+^CD16^-^RANK^hi^ cells can differentiate to osteoclasts when cells were exposed to RANKL. We further characterized CD14^+^CD16^–^RANK^hi^ cells by analyzing the expression of surface receptors. RANK^hi^ cells expressed CCR2, C3AR1 (GPCR of complement activation product 3a), CD51/CD61, and HLA-DR (Figure 2F). Hereafter, CD14^+^RANK^hi^ cells are referred to as cOCPs and CD14^+^RANK^–^ cells are called monocytes (MOs) (Supplemental Figure 4C). Our results indicate that a subset of CD14^+^ cells expressing RANK generates osteoclasts.

### cOCPs show distinct morphology and transcriptomic signatures.

**Figure 3:**
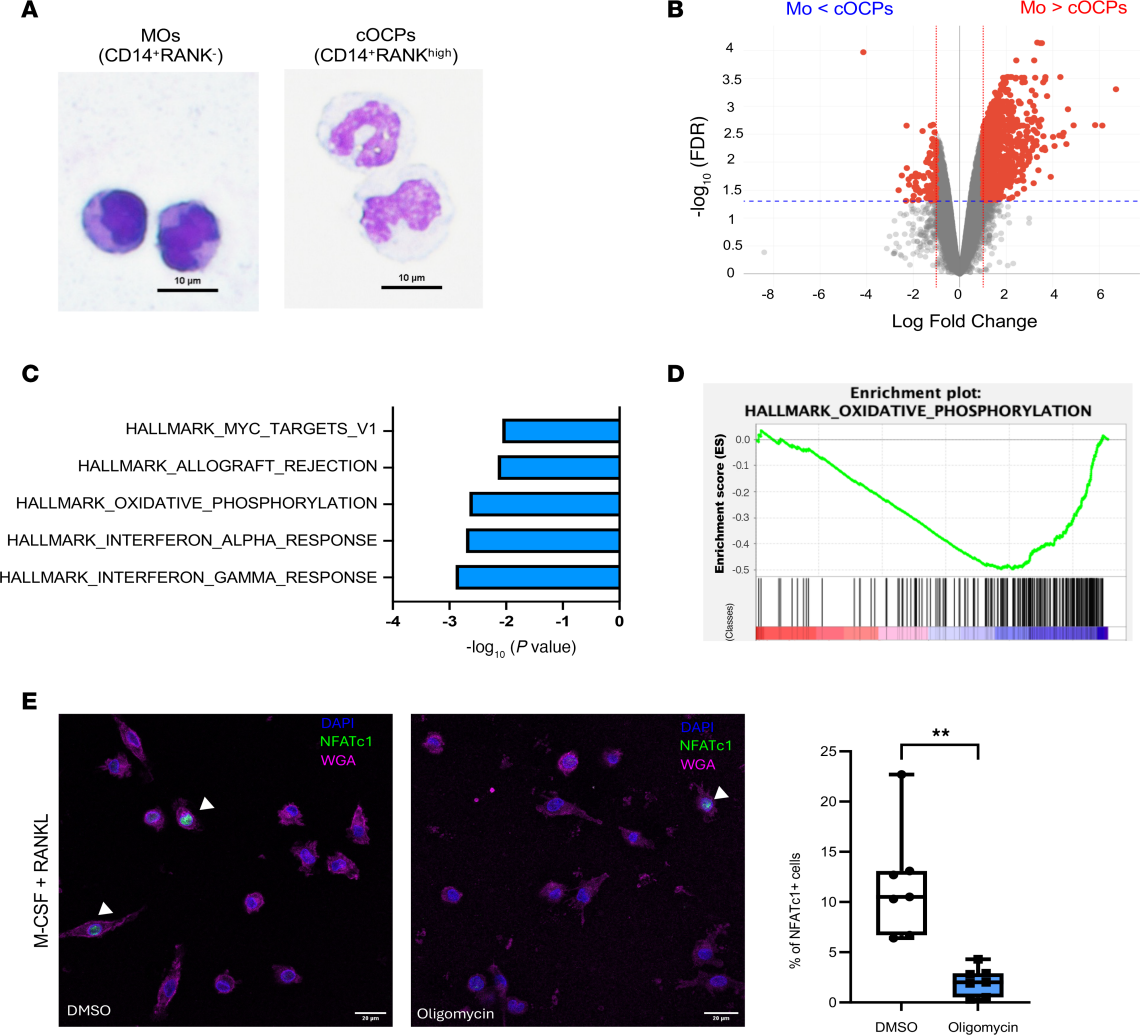
Circulating osteoclast-precursor cells have distinct morphology and transcriptomic signatures. (**A**) Representative images of Giemsa-stained monocytes (MOs) and osteoclast precursor cells (OCPs) that were sorted by FACS. Scale bar: 10 μm. (**B**) Volcano plot of RNA-Seq analysis of differentially expressed genes (DEGs) in OCPs and MOs. Significantly regulated genes (FDR < 0.01 and 2-fold change) are in red. (**C** and **D**) Gene set enrichment analysis (GSEA). (**C**) Top five canonical pathways by GSEA analysis. (**D**) The enrichment plot shows genes in the Hallmark oxidative phosphorylation gene set from the GSEA analysis. (**E**) Immunofluorescence staining with anti-NFTAc1 antibodies after culture for 1 day with RANKL. Cells were treated with 5 μM oligomycin or DMSO prior to RANKL stimulation. The left panel shows the representative images. The right panel shows quantification of NFATc1^+^ cells (*n* = 7). All data are shown as median and interquartile range. **P < 0.01 by 2-tailed Student’s *t* test.

To characterize cOCPs, we determined their cell morphology. cOCPs and MOs (RANK^–^CD14^+^ cells) were sorted by FACS analysis, and we then employed Giemsa staining (24). While MOs had a high nucleus-to-cytoplasm ratio, with typical monocyte or immature monocyte morphology, cOCPs exhibited a distinct morphology with floret nuclei (Figure 3A and Supplemental Figure 5). To gain an insight into the mechanism underlying the higher osteoclastogenic potential of cOCPs, we performed bulk RNA-Seq on sorted cOCPs and MOs. Owing to the limited number of cOCPs in each donor, samples included 2 biological replicates pooled from 11 independent donors. 963 genes were differentially regulated between cOCPs and MOs (FDR < 0.05 and >2-fold change) (Figure 3B). 104 genes were upregulated in cOCPs compared with MOs, whereas 859 genes were downregulated in cOCPs relative to MOs. Gene set enrichment analysis of differentially expressed genes showed that genes related to interferon responses, oxidative phosphorylation, and MYC targets were enriched in cOCPs (Figure 3, C and D). NADH:ubiquinone oxidoreductase core subunit S8 (NDUFS8) is an essential core subunit of mitochondrial complex I (25). NDUFS8 was one of the upregulated genes in cOCPs compared to MO and was confirmed by qPCR (Supplemental Figure 6). MYC regulates oxidative phosphorylation, which has a critical role in murine osteoclastogenesis (26). Consistent with murine data, RANKL treatment also enhanced the oxygen consumption rate of human CD14^+^ cells compared with M-CSF–alone conditions (Supplemental Figure 7, A–C). To test the role of oxidative phosphorylation in cOCPs, CD14^+^ cells were treated with oligomycin, a potent inhibitor for the mitochondria ATP synthase (27), prior to RANKL stimulation. While RANKL induced NFATc1 expression in a subset of CD14^+^ cells, oligomycin treatment significantly diminished NFATc1-expressing cells (Figure 3E) and osteoclastogenesis (Supplemental Figure 7D), supporting the importance of oxidative phosphorylation in osteoclastogenesis and in the function of cOCPs. Thus, our results indicate that cOCPs have distinct morphology and transcriptomic signatures.

### cOCPs significantly correlate with the bone density of postmenopausal women.

**Figure 4:**
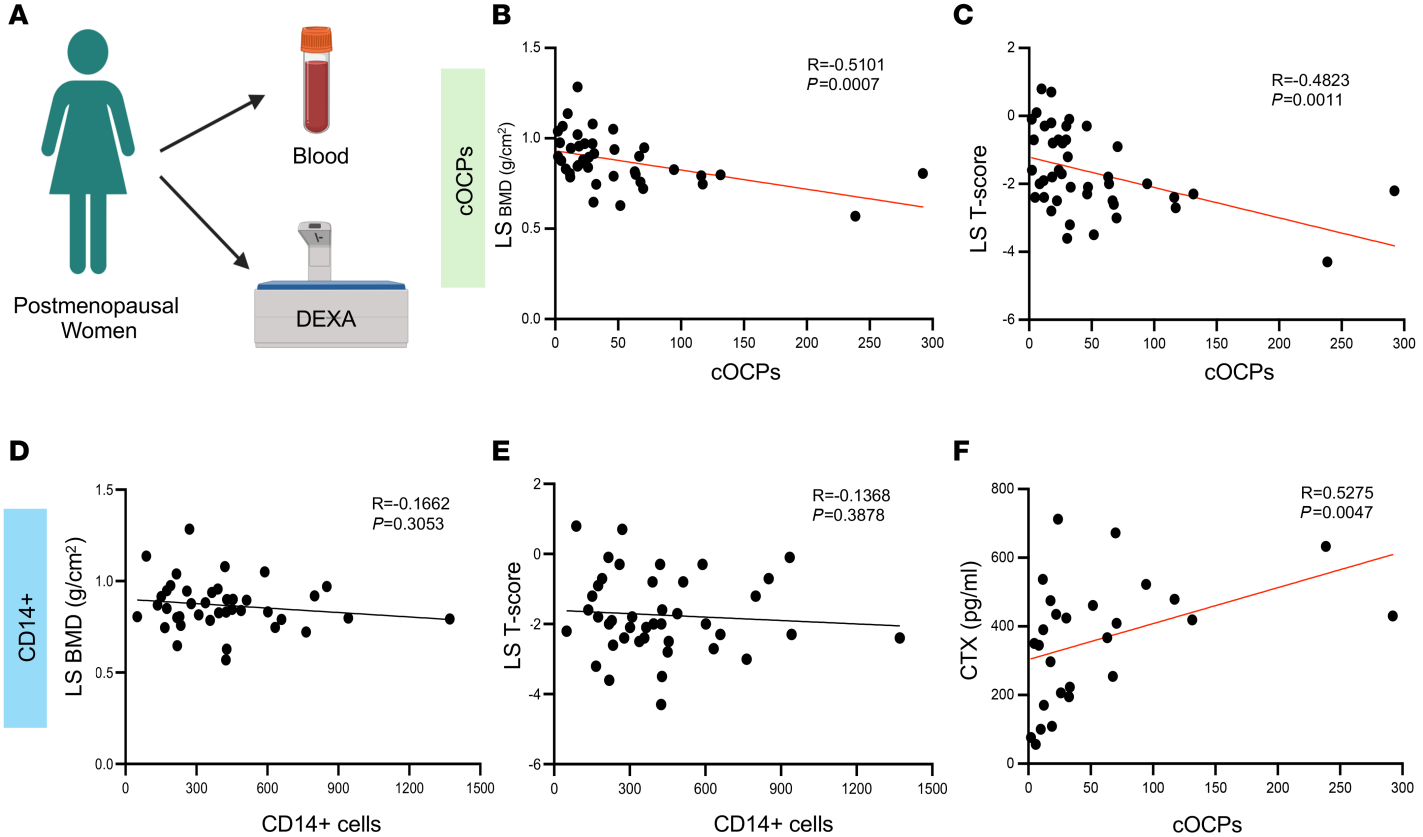
Circulating osteoclast precursor cells increase in patients with osteoporosis. (**A**–**E**) Bone density was determined by a DXA test, and cOCPs were numerated by flow cytometry in postmenopausal women (*n* = 44). (**A**) Schematic for experimental design. (**B** and **C**) A correlation plot between cOCPs and lumbar spine (LS) BMD (**B**) and lumbar spine T score (**C**). (**D** and **E**) A correlation plot between CD14^+^ monocytes and lumbar spine BMD (**D**) and lumbar spine T score (**E**). (**F**) A correlation plot between cOCPs and CTX. Spearman’s correlation test was used in **B**–**E**.

Since cOCPs travel back to bone and fuse with osteoclasts, we hypothesized that the frequency of cOCPs may correlate with osteoclast activity in vivo. To test this, we recruited 44 treatment-naive postmenopausal women; clinical information for the patients is listed in Table 1. Of the 44 individuals, 16 (36%) had osteoporosis, 19 (43%) had osteopenia, and 9 (20%) had normal BMD based on T scores. We quantified cOCPs in the blood by FACS analysis and analyzed bone density by DXA, respectively (Figure 4A). We found that the frequency of cOCPs was significantly inversely correlated with lumbar spine (LS) BMD values and the T score in all participants (*R* = −0.5101; *P* = 0.0007, *R* = −0.4823; *P* = 0.0011, respectively) (Figure 4, B and C), while CD14^+^ monocytes showed no association with LS BMD and T score (Figure 4, D and E). Moreover, postmenopausal women with osteoporosis (T score < –2.5) had significantly higher cOCPs than participants with normal bone density (T score ≥ –1) (Supplemental Figure 8A). However, the association between hip BMD and cOCPs was not significant (Supplemental Figure 8B).

We next measured the association between the frequency of cOCPs and bone turnover markers. The serum level of C-terminal type 1 collagen telopeptide (CTX), a bone resorption marker, was positively correlated with the number of cOCPs (*R* = 0.5275; *P* = 0.047) (Figure 4F), whereas serum levels of procollagen type I intact N-terminal propeptide (P1NP), bone formation markers, and RANKL did not show any correlation with the number of cOCPs (*R* = 0.1146; *P* = 0.4483 and *R* = 0.03982; *P* = 0.8027, respectively) (Supplemental Figure 8, C and D). Collectively, our results suggest that cOCPs may be closely related to the activity of osteoclasts in vivo.

### Denosumab treatment decreases the levels of cOCPs.

**Figure 5:**
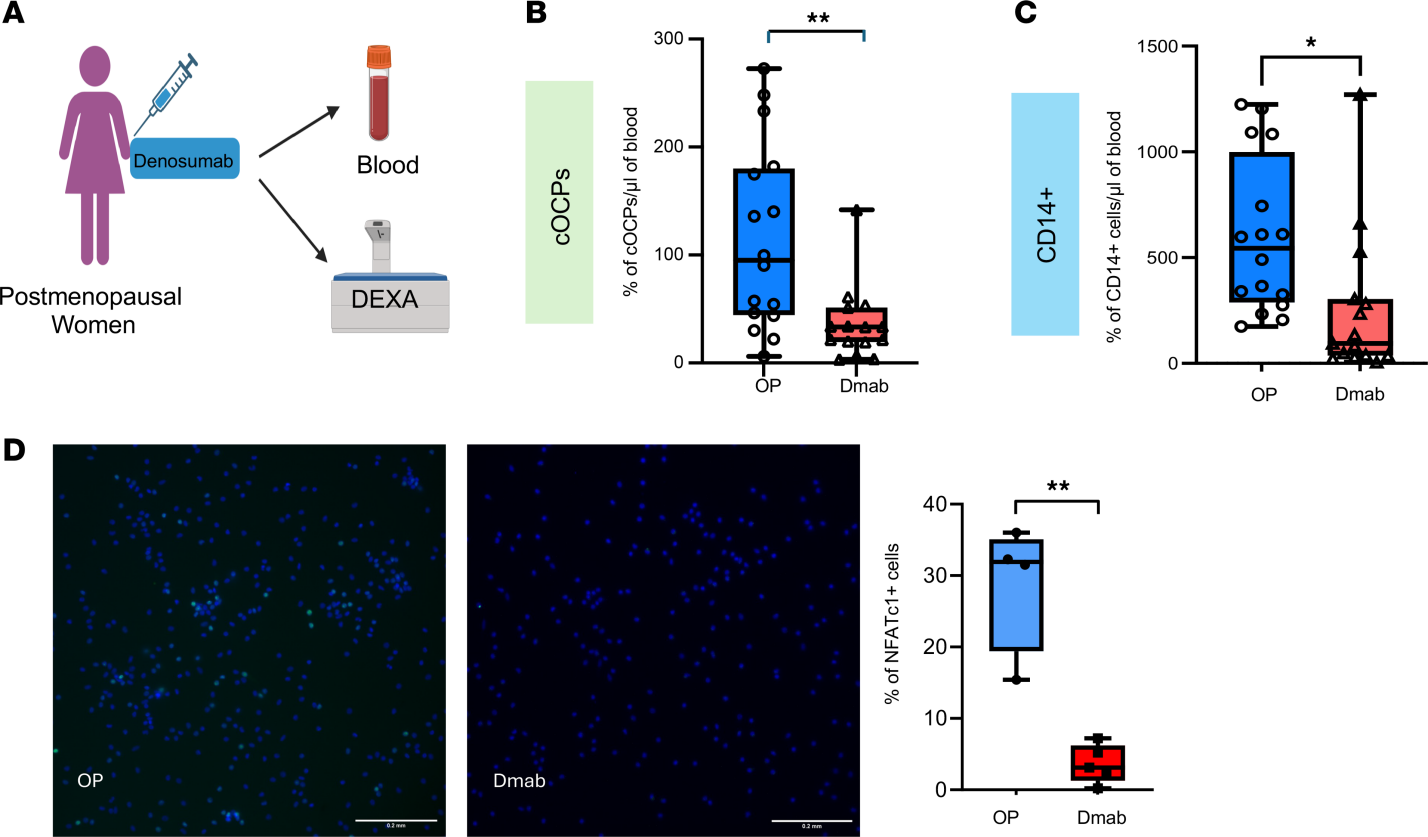
Circulating osteoclast precursor cells decrease in patients with osteoporosis treated with denosumab. (**A**) Schematic for experimental design. (**B** and **C**) A plot of the number of cOCPs (**B**) and CD14^+^ cells (**C**) in denosumab-treated patients (Dmab group, *n* = 15) and treatment-native patients with osteoporosis (OP, *n* = 16). (**D**) Immunofluorescence staining of DAPI and NFATc1. CD14^+^ cells from patients with osteoporosis and Dmab-treated patients were cultured with M-CSF and RANKL for 1 day. Then, the cells were stained with anti-NFATc1 antibodies. The left panels show representative images of DAPI (nucleus stain) and NFATc1 staining (*n* ≥ 4). Scale bar: 200 μm. The right panel shows the percentages of NFATc1^+^ cells per total cells. All data are shown as median and interquartile range. *P < 0.05; ** *P* < 0.01 by 2-tailed, unpaired *t* test in **B**–**D**.

Current antiresorptive strategies for targeting osteoclasts, such as denosumab, provide an efficacious treatment option for pathological bone resorption (28). Denosumab is an FDA-approved human monoclonal antibody against RANKL, which prevents RANK-mediated osteoclast formation (29). Denosumab treatment results in an increase in bone mass and has shown significant efficacy in reducing fracture risk in many bone diseases involving high osteoclast activity, such as osteoporosis (3, 30–32). To assess the relationship between the levels of cOCPs and denosumab treatment, we tested whether denosumab treatment can regulate the frequency of cOCPs. We measured the number of cOCPs in the blood of postmenopausal women who received denosumab treatment (referred to as the Dmab group). The median duration of denosumab treatment in our cohorts was about 5 years (Table 2). We compared the cOCPs between the individuals in the Dmab group and patients with osteoporosis from our postmenopausal cohorts (Figure 5A). The frequency of cOCPs in the Dmab group was significantly lower than that of treatment-naive patients with osteoporosis (Figure 5B). In addition, CD14^+^ monocytes, MOs, were diminished in the Dmab group compared with those in the osteoporosis group (Figure 5C). We measured NFATc1 immunofluorescence staining between the 2 groups. The same number of CD14^+^ cells from both groups was treated with M-CSF and RANKL for 1 day. NFATc1^+^ cells were significantly diminished in the Dmab group relative to treatment-naive patients with osteoporosis (Figure 5D), supporting our finding of a reduced number of cOCPs in the Dmab group. Our results suggest that denosumab treatment modulates the frequency of cOCPs in addition to its action on osteoclastogenesis.

## Discussion

In this study, we investigated a specific subset of circulating CD14^+^ cells named cOCPs and their association with BMD and CTX, a bone resorption marker, in postmenopausal women. Our study established that cOCPs are a distinct subset of CD14^+^ cells. cOCPs exhibited a high potential to differentiate into osteoclasts. Comparing the gene expression profile of cOCPs with other subsets of CD14^+^ monocytes, MOs, revealed notable differences, indicating that cOCPs show unique transcriptomic signatures. We also observed an inverse correlation between cOCPs and BMD and a positive association with CTX levels in postmenopausal women. While patients with osteoporosis had a significantly higher frequency of cOCPs, the number of cOCPs was diminished in patients with osteoporosis who received denosumab. Taken together, our study suggests that cOCPs can serve as a precursor to osteoclasts, which play an essential role in postmenopausal bone loss.

This is one of the first studies to our knowledge to establish a connection between cellular biomarkers and osteoporosis. Our study provides a platform for determining in vivo osteoclast activity by analyzing changes in blood cells. We found that the number of cOCPs was significantly higher in patients with osteoporosis compared with postmenopausal women with normal bone density. Intriguingly, cOCPs were correlated with CTX but not with bone formation markers such as P1NP, suggesting a correlation between cOCPs and osteoclast activity. Bone remodeling is a crucial process for maintaining healthy bones. Bone resorption exceeds bone formation and bone remodeling increases in patients with osteoporosis. It is well-established that both bone resorption markers and bone formation markers correlate with BMD (33). However, in postmenopausal women, bone formation markers did not decrease with age, while the rate of bone turnover in postmenopausal women is a major determinant of bone density (34). Ebeling et al. also showed that postmenopausal women had a greater level of bone turnover markers except for P1NP and free deoxypyridinoline (35). Thus, it may be possible that the lesser changes in bone formation markers compared with bone resorption markers lead to no significant correlation between cOCPs and bone formation markers in our study. We also found a significant correlation of cOCPs with LS BMDs but not with hip BMD. This discrepancy may result from the different bone compositional properties between the LS and femur bone. The LS has a high proportion of trabecular bone, resulting in active bone turnover, while the femoral neck has a high proportion of cortical bone, making it difficult to change bone density (36). Our findings may suggest that changes in cOCPs may be more closely linked to bone resorption in trabecular bone regions.

Our study demonstrated that human cOCPs have a distinct gene and protein expression pattern, making cOCPs a potential therapeutic target for bone diseases. Additionally, cOCPs have a unique morphology. cOCPs have a lower nuclear-to-cytoplasmic ratio than MOs and have an irregular nuclear shape. Furthermore, cOCPs seem to exhibit a mature phenotype, suggesting that blood CD14^+^ cells consist of cells at various stages. The risk factors and pathogenetic mechanisms causing osteoporosis have been studied extensively (37). Since OCPs can differentiate or fuse into osteoclasts, numerous studies investigated OCPs in bone marrow and blood (15). However, the majority of studies have been done in the murine system (11). It has been documented that human CD14^+^CD16^+^ cells serve as precursor cells for osteoclasts (38). However, the ability of CD16^+^ monocytes to differentiate into osteoclasts remains controversial (11). Our study also showed that CD16^+^ monocytes from healthy donors were not able to form osteoclasts. cOCPs express RANK, allowing cOCPs to respond to RANKL. Genes related to metabolic pathways, including genes related to oxidative phosphorylation, glycolysis, and fatty acid metabolism, were also enriched in cOCPs, suggesting that cOCPs may be metabolically active relative to monocytes. Metabolic reprogramming plays a crucial role in osteoclast formation and activity (39). Blocking oxidative phosphorylation inhibited the response to RANKL in cOCPs and human osteoclastogenesis, suggesting that cOCPs can be targeted directly for therapeutic purposes. OCPs also expressed chemokine receptors such as CCR2 and C3AR1. CCR2 is a chemokine receptor, and it plays an important role in monocyte recruiting. Genetic variants of CCR2 and MCP-1, a ligand for CCR2, have been suggested to correlate with osteoporosis (40). CCR2-deficient cells show defects in osteoclastogenesis and were prevented from ovariectomy-induced bone loss (41, 42). C-C motif chemokine ligand 2 (CCL2), a ligand of CCR2 is associated with low bone mass in postmenopausal women (43). C3AR1 is a central complement receptor for C3a (44). Mice deficient in C3AR1 exhibit higher trabecular bone density with increased osteoblast activity and reduced osteoclast number (45). Both CCR2 and C3AR1 positively regulate osteoclastogenesis, and their contribution to homing of cOCPs to bone needs to be characterized. Given the importance of cOCPs in generating osteoclasts, targeting cOCPs may serve as a new therapeutic strategy for pathological bone loss.

Denosumab is an effective antiresorptive medication, and it reduces bone loss and fracture risk (3, 30–32). Denosumab directly inhibits osteoclast differentiation by blocking the interaction of RANKL with RANK. Intriguingly, our study showed that denosumab treatment suppressed cOCPs, which may serve as an additional mechanism of denosumab’s action. In addition to cOCPs, CD14^+^ monocytes were diminished in the Dmab group compared with those in treatment-naive patients with osteoporosis. Several groups also studied the association of denosumab with CD14^+^ cells, relevant to MOs. A prospective open-label trial compared the effects of denosumab and zoledronate on CD14^+^CD11b^+^ cells and CD14^+^CD11b^+^VNR^+^ cells over 48 weeks in postmenopausal women with osteoporosis (46). In both denosumab and zoledronate-treated patients, CD14^+^CD11b^+^ cells decreased 48 weeks after treatment, while CD14^+^CD11b^+^VNR^+^ cells showed no significant changes (46). Kyrgidis et al. showed that both CD14^+^CD16^+^ and CD14^+^CD16^–^ populations were significantly decreased by denosumab administration at 48–72 hours after denosumab treatment, suggesting that denosumab treatment may modify monocyte populations (47). However, the reason why cOCPs and/or monocytes are reduced by denosumab treatment remains unclear. It is possible that the exit of cOCPs from the bone marrow to circulation may be blocked by denosumab treatment. Our results suggest that RANKL signaling may play a key role in bone marrow OCPs and monocytes traveling to the blood. Thus, in addition to direct blocking of osteoclast formation, denosumab treatment may reduce osteoclast activity and bone resorption by targeting cOCPs.

This study has some limitations. Its major limitation is that the research was conducted at a single hospital using a retrospective observational design. It also involved a relatively small study population and only included postmenopausal women. Additionally, we are unable to provide comments on the sequential changes in cOCPs over time. More large-scale prospective studies across multiple centers are necessary to support our conclusions. Despite the limitations of the study, our results suggest that the increase of cOCPs in the blood may reflect increased activity of osteoclasts. The detection of cOCPs, which is minimally invasive and requires a small blood sample, could be used to predict osteoclast activity and high-risk individuals for osteoporosis and help initiate diagnostic analysis and medical care for those who need it.

Altogether, our study demonstrates that a circulating cell population with high osteoclastogenic potential in human blood is a subset of monocytes and shows a strong correlation with bone density or a bone resorption marker in postmenopausal women. Our findings suggest that identifying increased precursor pools can be a valuable way to determine the trajectory of bone changes by increased osteoclast activity and provide evidence that cOCPs could circulate through blood and are affected by antiresorptive treatment.

## Methods

### Sex as a biological variable.

Our study examined postmenopausal women and healthy men and women who donated cells.

### Study population.

Participants included 44 postmenopausal women (mean age ± SD, 67.3 ± 9.2 years old) who visited the Endocrinology Service at the Hospital for Special Surgery. Of the 44 participants, 16 women (36%) had osteoporosis (mean age ± SD, 67.3 ± 8.8 years old), 19 (43%) had osteopenia (mean age ± SD, 66.8 ± 8.8 years old), and 9 (20%) had normal BMD (mean age ± SD, 66.1 ± 9.5 years old).

### Measurement of BMD.

Hip (total hip regions) and LS (L2–L4 region) BMD was evaluated using DXA on a Horizon A (Hologic). Information from the DXA scans, including T scores and BMD (measured in g/cm^2^), were obtained for the second through fourth lumbar vertebrae. T scores are used for the diagnosis of osteopenia or osteoporosis and are as follows: normal BMD, T score of –1 or higher; osteopenia, T score of –1 to –2.5; osteoporosis, T score of –2.5 or lower (8).

### Bone turnover markers.

Serum levels of the C-terminal telopeptides, β-cross-linked, serum (βCTX) were measured using ELECSYS β-CrossLaps/serum (ROCHE, 09005773190). P1NP was measured following the manufacturer’s instructions (Cloud-Clone Corp, SEA957Hu). RANKL was measured using Human RANKL DuoSet ELISA (R&D Systems, DY626).

### Osteoclast differentiation.

PBMCs from healthy participants were collected using Lymphoprep (Stem Cell Technologies) gradient centrifugation as previously described (15). Positive selection of monocytes was performed using CD14 MACS microbeads (Miltenyi Biotec) according to the protocol supplied by the manufacturer. Purified CD14^+^ cells were cultured with M-CSF (20 ng/mL, PeproTech) for 1 day in 96-well plates with αMEM (Thermo Fisher Scientific), 10% Hyclone fetal bovine serum (GE Healthcare Life Sciences), and 1% glutamine (200 mM, Thermo Fisher Scientific) and were further cultured for 3 days with M-CSF (20 ng/mL) and RANKL (40 ng/mL, PeproTech). Osteoclasts were fixed and stained for TRAP using the Acid Phosphatase Leukocyte diagnostic kit (Sigma-Aldrich) as recommended by the manufacturer. Multinucleated (>3 nuclei), TRAP^+^ osteoclasts were counted in triplicate wells.

### Flow cytometry.

PBMCs were prepared from blood, and red blood cells were lysed with ACK Lysing Buffer (Gibco Life Technologies). PBMCs were then incubated with antibodies (listed in Supplemental Table 1) at 4°C for 25 minutes, washed twice, resuspended in PBS containing 1% BSA, and analyzed on a FACS Canto II (BD Life Sciences) or FACS Symphony (BD Life Sciences). For cell sorting, CD14^+^RANK^+^CD66b^–^ cells were fractionated using a BD Influx Cell Sorter (BD Life Sciences) at the Weill Cornell Flow Cytometry Core.

### Cytospin preparation and staining.

For cytologic analysis of cell preparations, sorted 5,000 cells were mounted on slides using a Cytospin centrifuge for 5 minutes at 100*g*. Cells were air dried, fixed, and stained using a Giemsa stain (Sigma-Aldrich) according to the manufacturer’s instructions. Cytospin preparations were imaged using a standard light microscope using a ×40 magnification (Nikon). The morphology of cOCPs and MOs was reviewed by a pathologist from the Hospital for Special Surgery.

### Immunoblotting.

Whole-cell lysates were prepared using 1x Laemmli Sample Buffer (Bio-Rad) and were separated onto 7.5% or 10% SDS-PAGE, followed by standard Western blotting protocols. α-Tubulin (Sigma-Aldrich, T3026) was used as a loading control. NFATc1 antibody was purchased from BD Pharmingen (catalog 556602).

### Immunohistochemistry.

CD14^+^ cells (0.5 × 10^6^ cells/well) were allowed to adhere on 4-chamber slide well plates with M-CSF overnight and were further cultured with M-CSF and RANKL for 1 day. Cells were fixed in 4% paraformaldehyde for 20 minutes and treated with 0.2% Triton X for 5 minutes. Samples were washed 3 times with PBS, blocked in PBS with 1% (w/v) BSA (Sigma-Aldrich), 5% (v/v) nonspecific goat serum (MilliporeSigma), and 5% (v/v) nonspecific donkey serum (MilliporeSigma) for 20 minutes at room temperature. Samples were incubated with anti-NFATc1 antibody (1:200, Biolegend) overnight at 4°C. The next day the samples were washed and incubated with Alexa Fluor 488–conjugated anti-mouse IgG as secondary antibody (1:400, Jackson Immunoresearch) for 30 minutes at room temperature and then counterstained with DAPI (Invitrogen). Images were captured with a microscope.

### RNA-Seq.

Two biological replicates pooled from 11 independent donors (*n* = 5 and *n* = 6) were used for RNA-Seq. Total RNA was extracted using a RNeasy mini kit (Qiagen). True-seq RNA Library preparation kits (Illumina Inc.) were used to purify poly-A^+^ transcripts and generate libraries with multiplexed barcode adaptors following the manufacturer’s instructions. All samples passed quality control analysis on a Bioanalyzer 2100 (Agilent Technologies). Paired-end reads were obtained on an Illumina HiSeq 2500 in the Weill Cornell Medical College Genomics Resources Core Facility or the Weill Cornell Epigenomics Core Facility. Read quality was assessed with FastQC v0.11.6, and adapters were trimmed using Cutadapt v1.15. Reads were then mapped to the human genome (hg38), and reads in exons were counted against Gencode v27 with STAR Aligner v2.5.3a. Differential gene expression analysis was performed in R v3.5.1 using edgeR v3.20.9. Genes with low expression levels (<3 cpm in at least 1 group) were filtered from all downstream analyses. The Benjamini-Hochberg FDR procedure was used to calculate *q* values.

### Statistics.

Statistical analysis was performed with Prism version 8.0 software (Graphpad Software). Numerical data are expressed as both the mean ± SD and the median with the interquartile range. Welch’s *t* test was applied for numerical data when comparing 2 groups. Data with missing values were omitted automatically. Multiple comparisons were performed by 1-way ANOVA followed by Tukey’s multiple-comparison test or 2-tailed Student’s *t* test. Spearman’s rank correlation coefficient test was employed to evaluate the correlation of assessed parameters. Two-tailed Student’s *t* test or Welch’s *t* test was applied for numerical data when comparing 2 groups. A power analysis was performed using G*Power 3.1 (48). *P* values of less than 0.05 were considered statistically significant.

### Study approval. Recruited participants provided written informed consent.

This study was approved by the Hospital for Special Surgery’s Institutional Review Board (IRB 2016–0663).

### Data availability.

The RNA-Seq data have been deposited in the Gene Expression Omnibus database with the accession code GSE276973. Values for all data points in the graphs can be found in the Supporting Data Values file.

## Author contributions

KK curated data; provided formal analysis, validation, investigation, visualization, methodology, and resources; wrote the original draft of the manuscript; and reviewed and edited the manuscript. JT provided formal analysis and investigation; curated data; and reviewed and edited the manuscript. DM provided formal analysis; curated data; and reviewed and edited the manuscript. BO curated data and provided investigation. AJS, SB, MM, and AU curated data. EG and TF provided bioinformatic analysis. YZ provided a pathology analysis. EMS provided validation and methodology and reviewed the manuscript. RSB provided resources, supervision, validation, and methodology and reviewed and edited the manuscript. KHPM provided conceptualization, resources, supervision, and validation; curated data; acquired funding; and reviewed and edited the manuscript.

## Supplementary Material

Supplemental data

Unedited blot and gel images

Supporting data values

## Figures and Tables

**Table 1:**
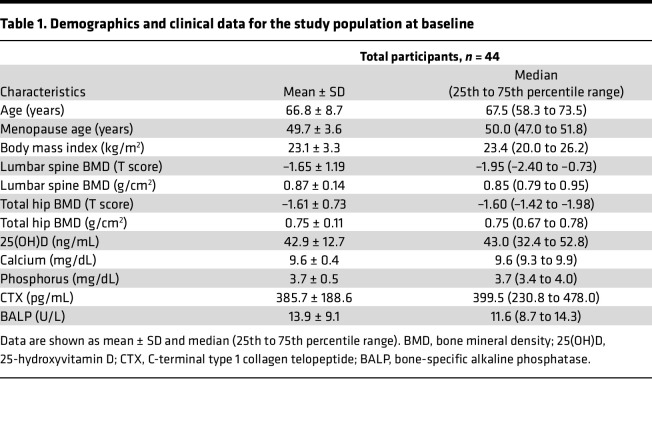
Demographics and clinical data for the study population at baseline

**Table 2:**
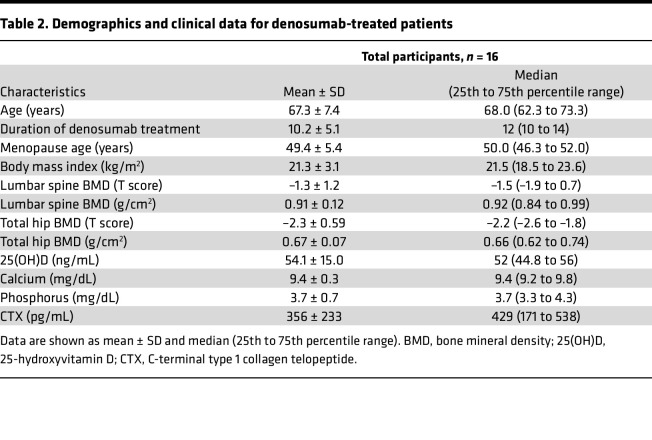
Demographics and clinical data for denosumab-treated patients

## Version 1 11/22/2024

Electronic publication
