# Supplementary material for: Cellular signatures in human blood track bone mineral density in postmenopausal women
Source: JCI Insight. 2024 Nov 22;9(22):e178977. doi: 10.1172/jci.insight.178977 (PMC11601907; doi:10.1172/jci.insight.178977)
Supplement: Supplemental data [file jciinsight-9-178977-s271.pdf]

Supplemental information

Cellular signatures in human blood track bone mineral density in postmenopausal women

Kaichi Kaneko, Jefferson Tsai, Deniece Meñez, Oh Brian, Andrew Junwoo Suh, Seyeon Bae, Masataka Mizuno, Akio Umemoto, Eugenia Giannopoulou, Takayuki Fujii, Yaxia Zhang, Emily Stein, Richard Bockman, and Kyung-Hyun Park-Min

	Pages
Supplemental Figures (1-8)	2-9
Supplemental Figure Legends	10-11
Supplemental Methods	12
Supplemental Table 1	13

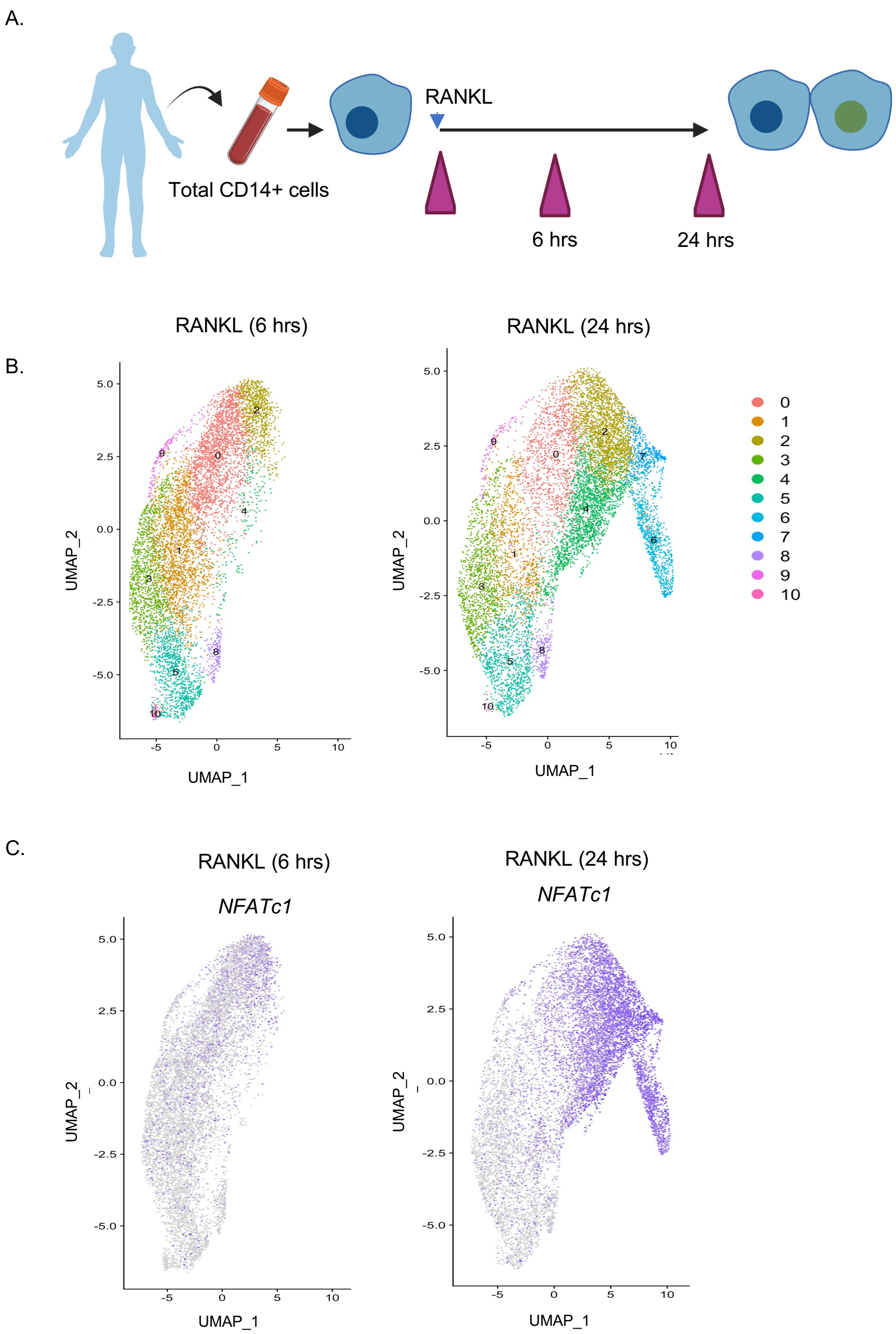
A.
Total CD14+ cells
RANKL
6 hrs
24 hrs
B.
RANKL (6 hrs)
RANKL (24 hrs)
UMAP_2
UMAP_1
0
1
2
3
4
5
6
7
8
9
10
C.
RANKL (6 hrs)
RANKL (24 hrs)
NFATc1
NFATc1
UMAP_2
UMAP_1

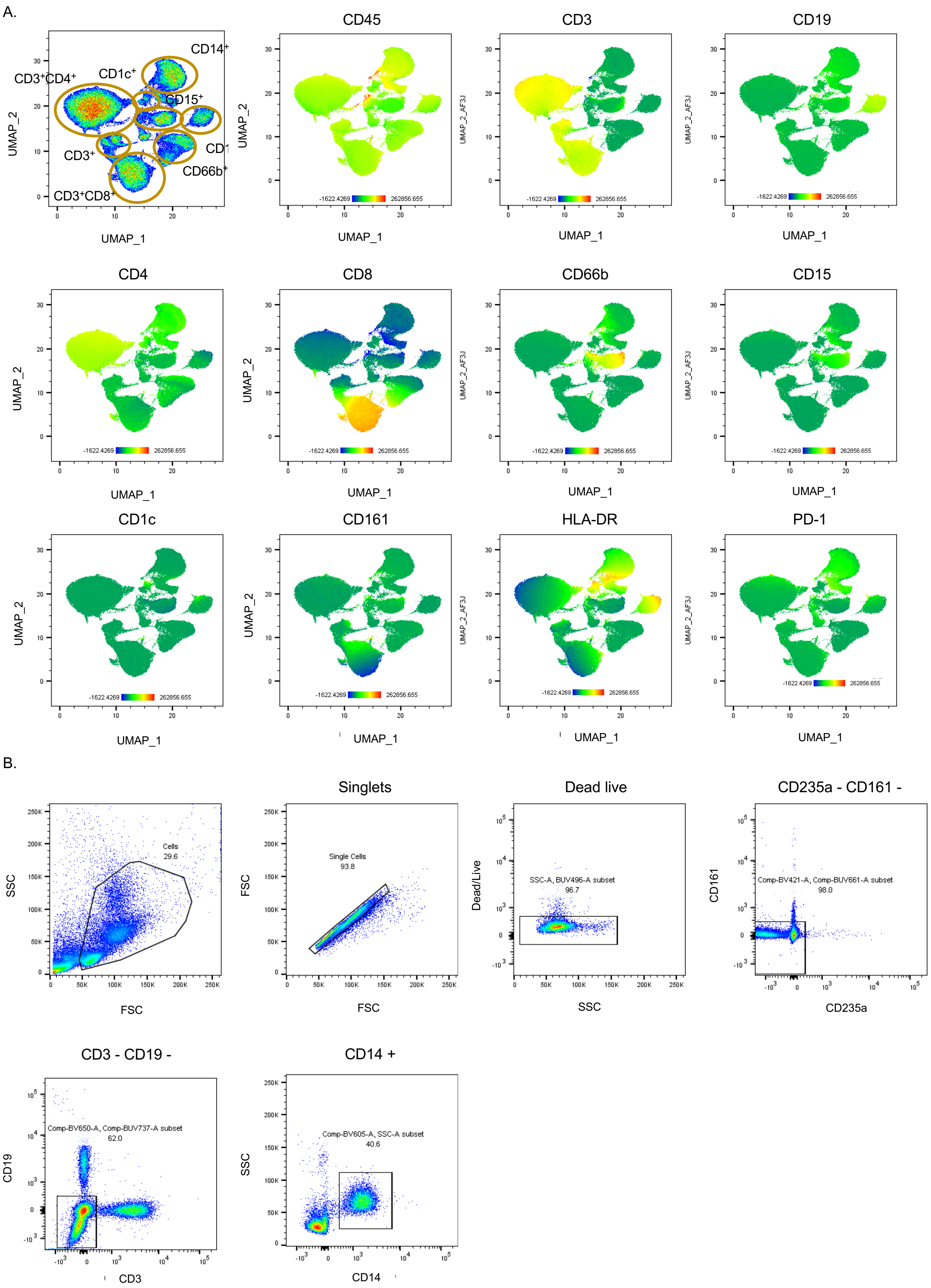
A.
CD14+
CD1c+
CD3+CD4+
CD15+
CD3+
CD66b+
CD3+CD8+
UMAP_1
UMAP_2
CD45
CD3
CD19
CD4
CD8
CD66b
CD15
CD1c
CD161
HLA-DR
PD-1
UMAP_2_AF3J
-1622.4269
262856.655
B.
Cells
29.6
SSC
FSC
Singlets
Single Cells
93.8
Dead live
SSC-A, BUV496-A subset
96.7
Dead/Live
CD235a - CD161 -
Comp-BV421-A, Comp-BUV661-A subset
98.0
CD161
CD235a
CD3 - CD19 -
Comp-BV650-A, Comp-BUV737-A subset
62.0
CD19
CD3
CD14 +
Comp-BV605-A, SSC-A subset
40.6
CD14

A.

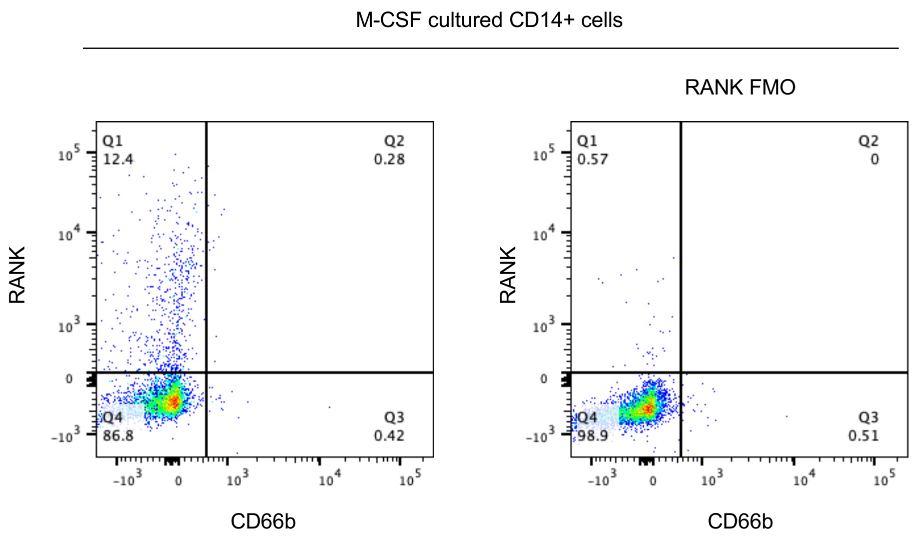
M-CSF cultured CD14+ cells
RANK FMO
RANK
CD66b
Q1
12.4
Q2
0.28
Q3
0.42
Q4
86.8
Q1
0.57
Q2
0
Q3
0.51
Q4
98.9

B.

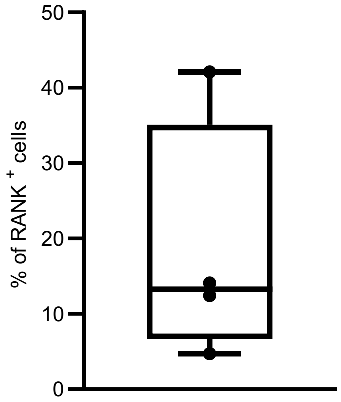
% of RANK + cells
0
10
20
30
40
50

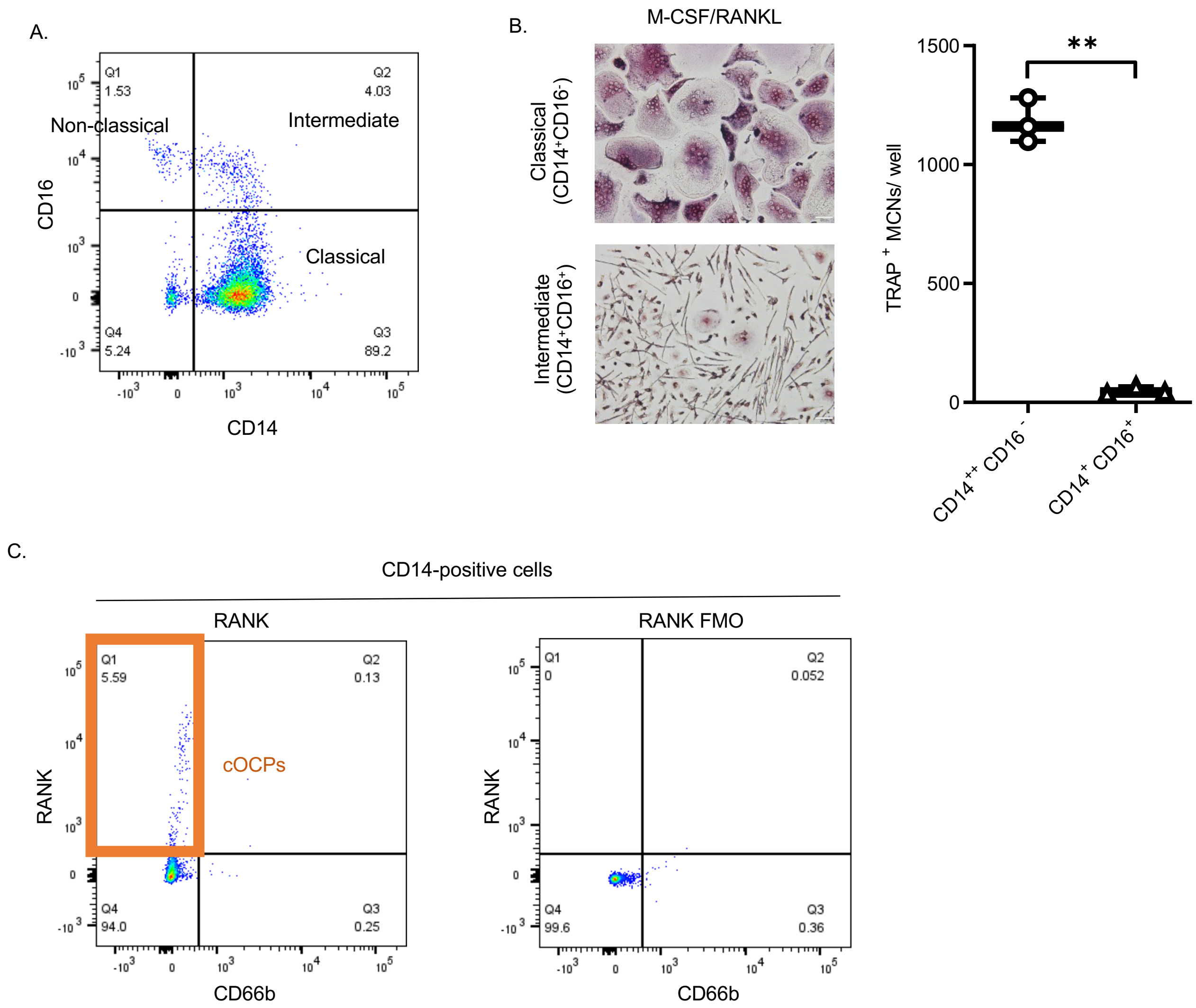

A.
Q1
1.53
Q2
4.03
Non-classical
Intermediate
Classical
Q4
5.24
Q3
89.2
CD16
CD14
B.
M-CSF/RANKL
Classical (CD14+CD16-)
Intermediate (CD14+CD16+)
TRAP + MCNs/ well
1500
1000
500
0
**
CD14++ CD16 -
CD14+ CD16+
C.
CD14-positive cells
RANK
Q1
5.59
Q2
0.13
cOCPs
Q4
94.0
Q3
0.25
CD66b
RANK FMO
Q1
0
Q2
0.052
Q4
99.6
Q3
0.36
CD66b

A.

MOs
($CD14^+$ $RANK^-$)

cOCPs
($CD14^+$ $RANK^{high}$)

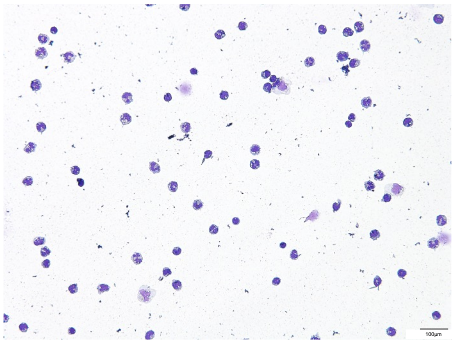

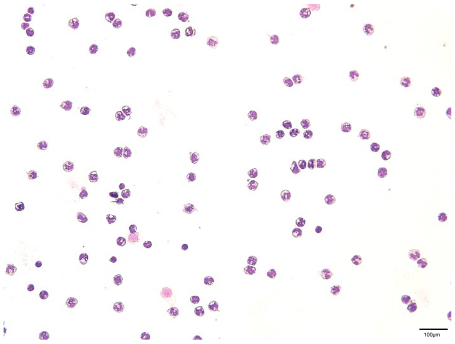

B.

MOs
($CD14^+$ $RANK^-$)

cOCPs
($CD14^+$ $RANK^{high}$)

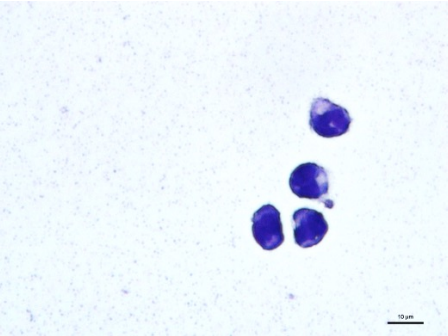

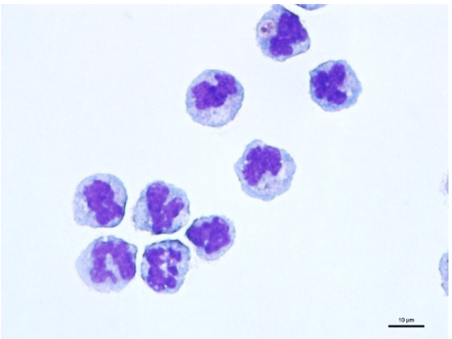

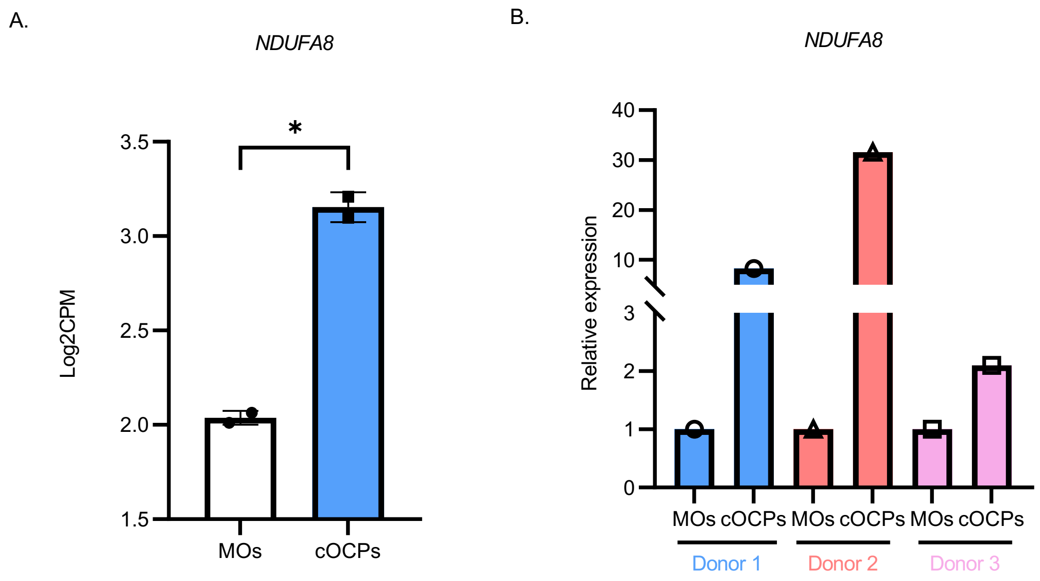

A.
NDUFA8
*
3.5
3.0
2.5
2.0
1.5
Log2CPM
MOs
cOCPs
B.
NDUFA8
40
30
20
10
3
2
1
0
Relative expression
MOs cOCPs MOs cOCPs MOs cOCPs
Donor 1
Donor 2
Donor 3

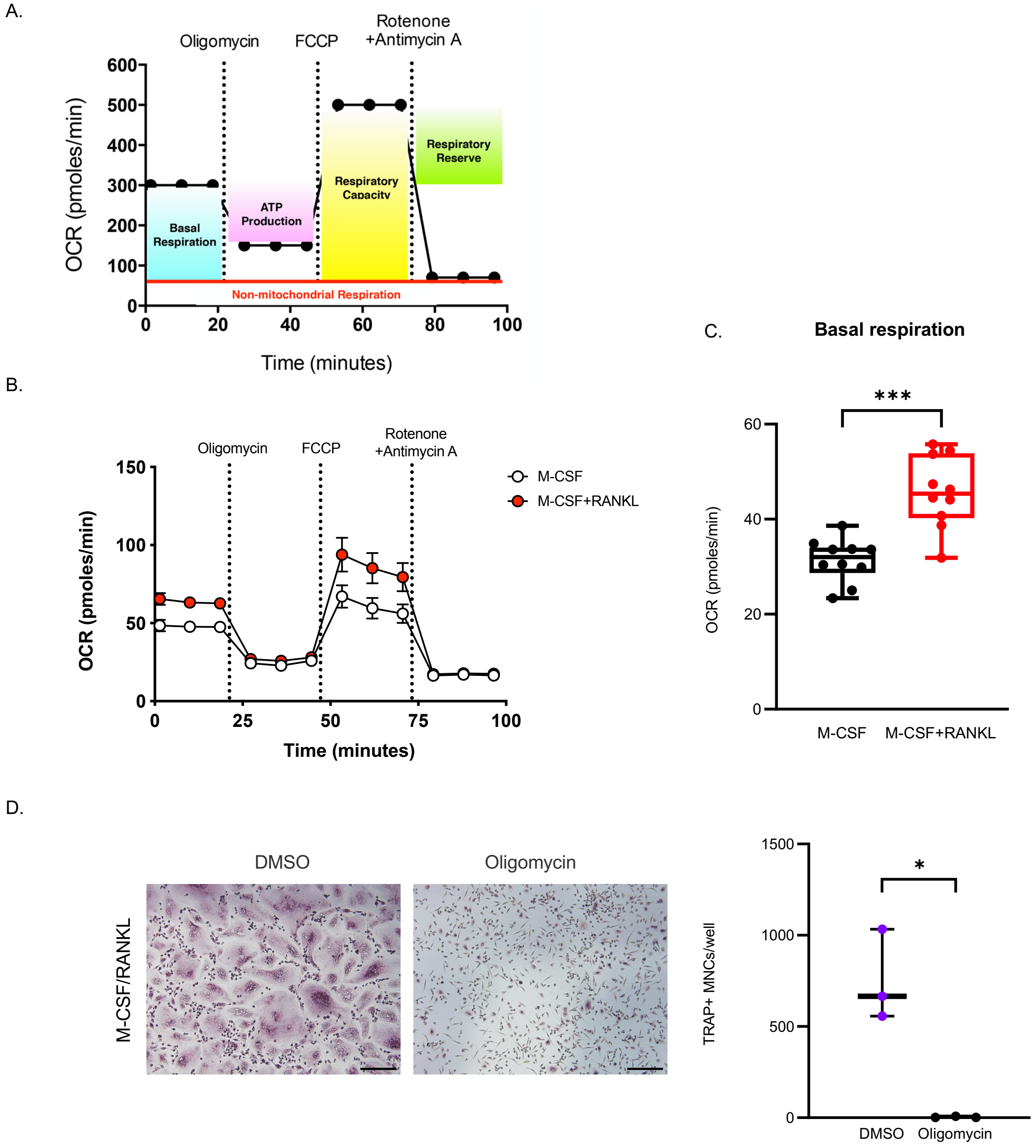

A.
Oligomycin
FCCP
Rotenone
+Antimycin A
600
500
400
300
200
100
0
OCR (pmoles/min)
Basal
Respiration
ATP
Production
Respiratory
Capacity
Respiratory
Reserve
Non-mitochondrial Respiration
0
20
40
60
80
100
Time (minutes)
B.
Oligomycin
FCCP
Rotenone
+Antimycin A
M-CSF
M-CSF+RANKL
150
100
50
0
OCR (pmoles/min)
0
25
50
75
100
Time (minutes)
C.
Basal respiration
***
60
40
20
0
OCR (pmoles/min)
M-CSF
M-CSF+RANKL
D.
DMSO
Oligomycin
M-CSF/RANKL
1500
1000
500
0
TRAP+ MNCs/well
*
DMSO
Oligomycin

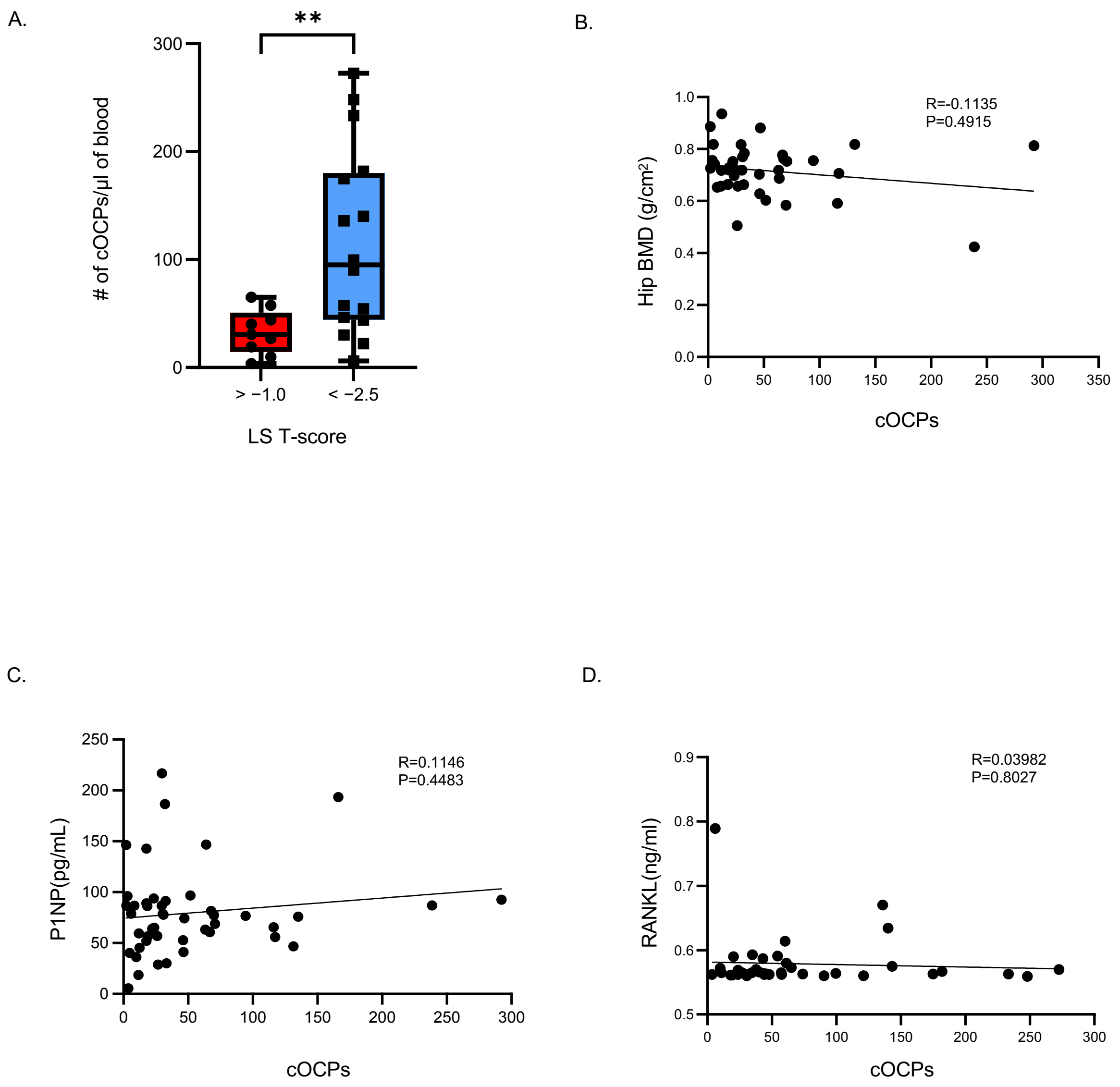
A.
**
300
200
100
0
# of cOCPs/µl of blood
> −1.0
< −2.5
LS T-score
B.
R=-0.1135
P=0.4915
Hip BMD (g/cm²)
1.0
0.8
0.6
0.4
0.2
0.0
0
50
100
150
200
250
300
350
cOCPs
C.
R=0.1146
P=0.4483
P1NP(pg/mL)
250
200
150
100
50
0
0
50
100
150
200
250
300
cOCPs
D.
R=0.03982
P=0.8027
RANKL(ng/ml)
0.9
0.8
0.7
0.6
0.5
0
50
100
150
200
250
300
cOCPs

Supplemental Figure Legends

Supplemental Figure 1. A subset of CD14+ cells responds to RANKL. CD14+ cells were isolated from human blood. Cells were cultured with M-CSF for one day and then stimulated with RANKL for 6 and 24 hours. A. Schematic of the experimental design. B. UMAP analysis of CD14+ cells. C. UMAP feature plot showing NFATc1 expression (Purple).

Supplemental Figure 2. UMAP analysis of human PBMCs. PBMCs from healthy donors (n=9) were stained with 16 antibody markers and analyzed by flow cytometry analysis. A. UMAP plot showed for each antibody-enriched population. B. Gating strategy to identify CD14+ cells.

Supplemental Figure 3. RANK expression. CD14+ cells were cultured with M-CSF for one day and RANK expression was analyzed by flow cytometry. A. Representative flow plots for M-CSF- cultured cells. RANK Fluorescence Minus One (FMO) used all antibodies except the anti-RANK antibody. B. Quantification of the percentage of RANK-positive cells (n=4). All data are shown as median and interquartile range.

Supplemental Figure 4. $CD14^{+}CD16^{+}$ cells do not differentiate into osteoclasts. A-B. PBMCs were analyzed by flow cytometry. Monocytes are classified as classical ($CD14^{+}CD16^{-}$), intermediate ($CD14^{+}CD16^{+}$), and non-classical ($CD14^{-}CD16^{+}$). (n=3) A. Representative flow plot. B. Osteoclastogenesis assay. Left Panel: Representative images of TRAP-stained cells. Right panel: Quantification of TRAP+ multinuclear osteoclasts. All data are shown as median and interquartile range. ***; p<0.005 by Student t-test. C. Representative flow plots for human PBMCs. RANK Fluorescence Minus One (FMO) used all antibodies except RANK antibodies. Among the CD14+ population, RANK high/CD66b-negative cells are named circulating osteoclast-precursor cells (cOCPs), and RANK negative/CD66b-negative cells are called monocytes (MOs).

Supplemental Figure 5. Giemsa-stained monocytes (MOs) and circulating osteoclast precursor cells (cOCPs) that are sorted by FACS. A. The images of lower resolution of Figure 3A. B. Representative images of cell morphology from another donor.

Supplemental Figure 6. The expression of NDUFA8. A. Log2 CPM value of NFUFA8 mRNA from RNA sequencing data pooled from 11 independent donors. B. mRNA expression of NDUFS8 in sorted cOCPs and MOs was analyzed by qPCR. MOs and cOCPs were sorted from three independent donors.

Supplemental Figure 7. Oxidative phosphorylation plays an important role in human osteoclastogenesis. A. A method of obtaining individual components of oxygen consumption rate (OCR). After injecting inhibitors that affect mitochondrial function, real-time OCR measurement was completed. The area under the curve of the resulting graph was used to calculate Basal Respiration, ATP Production, Respiratory Capacity, and Respiratory Reserve, according to the manufacturer's protocol. B, C $CD14^{+}$ cells were cultured with RANKL for two days. Mitochondrial function was assessed by real-time OCR measurement after sequential treatment of compounds modulating mitochondrial function (n=10). The OCR was normalized to the relative amount of DNA. B. Representative time course data. C. Assessment of mitochondrial activity. D. Osteoclastogenesis assay. CD14+ cells were treated with 5μM of oligomycin prior to RANKL stimulation (n=3). Cells were treated with RANKL for three days. Left panel: Representative images. Right panel: Quantification of TRAP+ multinuclear osteoclasts. All data are shown as median and interquartile range. ****;p<0.001, *; p<0.05, by Student t-test.

Supplemental Figure 8. cOCPs are higher in osteoporosis patients than postmenopausal women with normal bone density All the values are obtained from the patients used in Figures 4 and 5. A. The frequency of cOCPs in postmenopausal women with normal bone density (Lumbar Spine (LS) T score > -1.0, n=9) or osteoporosis patients (LS T score <-2.5, n=16) from Figure 5B. *; $p<0.05$, by Student t-test. B. Correlation plot between Hip BMD and cOCPs. C. Correlation plot between P1NP and cOCPs. D. Correlation plot between RANKL and cOCPs. Spearman correlation test in B-D.

Supplemental Methods

Single-cell RNA sequencing library preparation and data processing CD14+ cells were loaded onto the Chromium Controller using Single Cell 3' v3 Reagent following the manufacturer's protocol (10× Genomics, Pleasanton, CA, USA). Paired-end reads were obtained on an Illumina HiSeq 4000 in the Weill Cornell Medical College Genomics Resources Core Facility. Reads were mapped and reads in exons were counted to the human genome (GRCh38) using CellRanger v3.0. Cells with gene number < 300 and mitochondrial gene > 95 percentile were filtered out from each dataset. After filtering, scaling normalization by deconvolving size factors was performed by Scran v1.12.1, and doublets were excluded using scDblFinder v1.0. The gene count matrix of all datasets was integrated with Seurat v.3 to remove batch effects across different samples. In parameter settings, the first 40 dimensions of canonical correlation analysis (CCA) were used. Statistical inference of principal-component analysis (PCA) was calculated by the JackStraw function of Seurat, and the first 40 dimensions of principal components were used for dimensionality reduction by UMAP. Clustering was performed using k-nearest Neighbors graph construction and Louvain community detection. The RNA-seq data have been deposited in the Gene Expression Omnibus database with the accession code GSE276768.

Mitochondrial oxygen consumption measurement. The real-time mitochondrial OCR and extracellular acidification rate (ECAR) were measured using the XF96 Extracellular Flux Analyzer (Seahorse Bioscience) with the Cell Mito Stress Kit (Seahorse Bioscience) following the manufacturer's instructions. The measurement was normalized to the relative level of DNA determined by measuring the fluorescence intensity of cells stained by SYTO 24 Green Fluorescent Nucleic Acid Stain (Molecular Probes). Briefly, CD14+ cells from healthy donors were seeded on XF96 cell culture microplates (Seahorse Bioscience) at a seeding density of 8 Å~ 103 cells per well and stimulated with RANKL (50 ng/ml) for 2 days. Before the assay, cells were rinsed twice and kept in a prewarmed XF assay medium (pH 7.4) supplemented with 1 mM sodium pyruvate, 2 mM glutamine, and 10 mM glucose in a 37℃ non-CO2 incubator for 1 hour. Next, the respiratory rate was measured at 37℃ in at least 3 replicates per condition by using the following perturbation drugs in succession: 1 μM oligomycin, 2 μM carbonyl cyanideptrifluoromethoxyphenyl-hydrazone (FCCP), and 0.5 μM rotenone/antimycin A. The basal OCR was measured before drug exposure. We calculated the mitochondrial function metrics as described in Supplemental Figure S4 and as directed in the Cell Mito Stress Kit manual (Seahorse Bioscience).

Supplemental Table 1. Antibodies used for the study

Cluster designation	Clone	Company
CD1c	F10/21A3	BioLegend
CD3	OKT3	BioLegend
CD4	SK3	BioLegend
CD8	SK1	BioLegend
CD14	M5E2	BioLegend
CD16	3G8	BioLegend
CD19	SJ25C1	BD Horizon
CD51/CD61	23C6	BioLegend
CD15	BV711	BioLegend
CD66b	G10F5	BioLegend
CD161	DX12	BD Biosceince
CD235a	HI264	BioLegend
C3AR1	hC3aRZ8	BioLegend
CCR2	K036C2	BioLegend
HLA-DR	G46-6	BD Biosceince
RANK	9A725	Novus Biological
