# Supplementary figures and images for: Cellular signatures in human blood track bone mineral density in postmenopausal women
Source: JCI Insight. 2024 Nov 22;9(22):e178977. doi: 10.1172/jci.insight.178977 (PMC11601907; doi:10.1172/jci.insight.178977)

**Fig 1B. *Unedited blot and gel images***

**NFATc1**

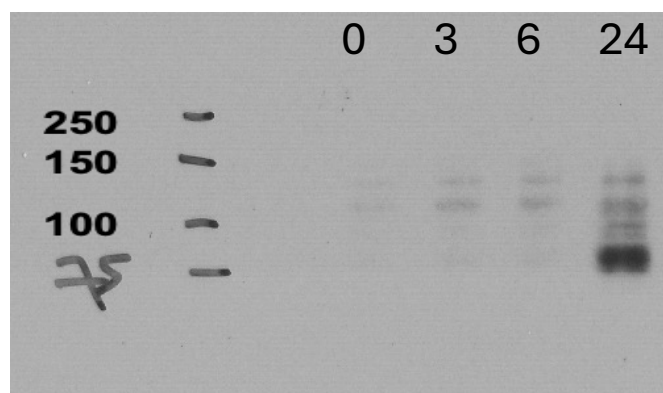

**$\alpha$ -tubulin**

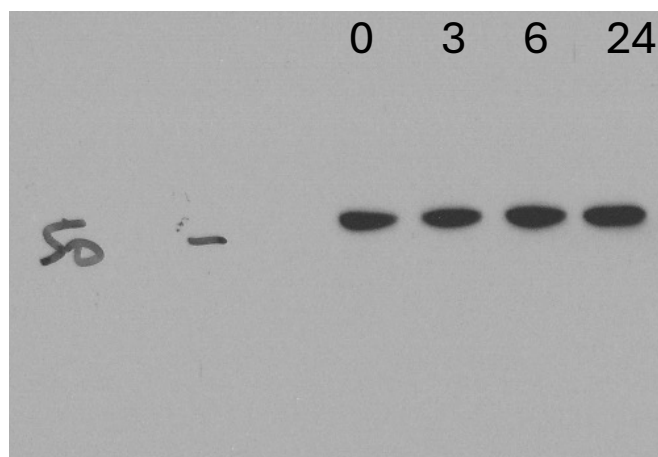

Supplement: Unedited blot and gel images [file jciinsight-9-178977-s272.pdf]
